# Automatic segmentation of liver structures in multi-phase MRI using variants of nnU-Net and Swin UNETR

**DOI:** 10.1038/s41598-025-07084-5

**Published:** 2025-07-16

**Authors:** Florian Raab, Quirin Strotzer, Christian Stroszczynski, Claudia Fellner, Ingo Einspieler, Michael Haimerl, Elmar W. Lang

**Affiliations:** 1 Physics Department, University of Regensburg, 93053 Regensburg, Germany; 2 Biophysics Department, CIML Group, University of Regensburg, 93053 Regensburg, Germany; 3 Radiology Department, University Hospital Regensburg, 93053 Regensburg, Germany; 4 Radiology Department, Klinikum Würzburg Mitte, 97074 Würzburg, Germany

**Keywords:** Liver, Liver diseases, Machine learning, Magnetic resonance imaging

## Abstract

Accurate segmentation of the liver parenchyma, portal veins, hepatic veins, and lesions from MRI is important for hepatic disease monitoring and treatment. Multi-phase contrast enhanced imaging is superior in distinguishing hepatic structures compared to single-phase approaches, but automated approaches for detailed segmentation of hepatic structures are lacking. This study evaluates deep learning architectures for segmenting liver structures from multi-phase Gd-EOB-DTPA-enhanced T1-weighted VIBE MRI scans. We utilized 458 T1-weighted VIBE scans of pathological livers, with 78 manually labeled for liver parenchyma, hepatic and portal veins, aorta, lesions, and ascites. An additional dataset of 47 labeled subjects was used for cross-scanner evaluation. Three models were evaluated using nested cross-validation: the conventional nnU-Net, the ResEnc nnU-Net, and the Swin UNETR. The late arterial phase was identified as the optimal fixed phase for co-registration. Both nnU-Net variants outperformed Swin UNETR across most tasks. The conventional nnU-Net achieved the highest segmentation performance for liver parenchyma (DSC: 0.97; 95% CI 0.97, 0.98), portal vein (DSC: 0.83; 95% CI 0.80, 0.87), and hepatic vein (DSC: 0.78; 95% CI 0.77, 0.80). Lesion and ascites segmentation proved challenging for all models, with the conventional nnU-Net performing best. This study demonstrates the effectiveness of deep learning, particularly nnU-Net variants, for detailed liver structure segmentation from multi-phase MRI. The developed models and preprocessing pipeline offer potential for improved liver disease assessment and surgical planning in clinical practice.

## Introduction

Liver volumetry, relying on anatomically accurate structural segmentations, plays an increasingly important role in the assessment of liver disease. Applications include delineating liver segments of interest in planned partial liver resection, measuring volumes of liver tissue in procedures that aim to induce segment hypertrophy, or monitoring tumor progress^1^. The planning of procedures in interventional oncology, like microwave ablation or selective internal radiation therapy, is increasingly benefiting from the segmentation of anatomical structures like tumors and vessels as well. Furthermore, applications of artificial intelligence in medical imaging regularly rely on segmented structural images, e.g., in radiomics, where quantitative biomarkers are extracted from imaging, for example, to identify microvascular invasion in hepatocellular carcinoma^2^.

Most applications in research and clinical practice employ a manual approach to the segmentation of volumes of interest. In a recent review focusing on precision medicine in hepatocellular carcinoma, Wei et al. reported that 82.1% of published studies were based on manual segmentations^3^. Manual approaches are time-intensive and susceptible to inter-rater variability. Furthermore, automatic segmentation of the liver was proven preferable to the manual placement of regions of interest, e.g., for the assessment of steatosis and iron quantification in chronic liver disease^4^. Hence, the development of automated approaches has received considerable attention in recent years and consequently resulted in the use of deep learning methods.

The most frequently used architectures today are based on U-shaped convolutional neural networks. These were introduced by Ronneberger et al. in 2015 and rely on an encoder-decoder design^5^. One of the most widely used adoptions to date is the nnU-Net, a self-configuring framework optimized for medical image segmentation^6^. More recently, transformer-based architectures that rely on a self-attention mechanism have been gaining popularity, whereby the SwinUNETR particularly stands out^7^. Moreover, models based on the newly introduced Mamba architecture that incorporates a structured state space sequence model were just released^8^. Most applications employ automated segmentation of the liver and adjacent structures based on CT data. Advantages are the wide availability, the good contrast of abdominal structures, and the standardized Hounsfield scale.

Segmentation based on MRI has been less widely employed but is of equal importance, especially since it doesn’t use any harmful radiation. Studies primarily focus on whole organ or tumor segmentation. However, much more detailed segmentations would be possible based on the wealth of information about tissue properties obtained with dynamically contrast enhanced MRI. Several studies have successfully evaluated liver function using Gd-EOB-DTPA (Primovist^®^, Bayer Healthcare, Berlin, Germany) -enhanced T1-volumetric interpolated breath-hold examination (VIBE) MRI sequences. However, measurements were performed with manually placed ROIs, either in liver parenchyma areas, explicitly excluding lesions and vascular structures, or in vascular structures excluding liver parenchyma, as well as in the abdominal aorta^9–14^. This is problematic, since other studies demonstrate that the liver function is not always homogeneously distributed in the liver^15–17^ and global assessments via blood or breath tests fail to capture localized variations. On top of that, only a few studies address the segmentation of liver vasculature using deep learning techniques, at all^18–20^.

However, neither code nor trained models are available, substantially limiting the possible benefits of the proposed works.

To address the limitations of manual segmentations for pre-operative planning and non-automated ROI placements for liver function assessment, we aimed to develop a model capable of providing automated and detailed anatomical segmentations of the liver and associated structures. Additionally, we sought to make the trained models and the entire preprocessing pipeline publicly available to maximize their utility and aid other research in this area. We opted for the nnU-Net, as it has been extensively tested and has proven effective in medical segmentation tasks. This was reaffirmed in recent comparisons with more complex and advanced architectures^21^. We also tested a more recent Vision Transformer architecture, where the Swin UNETR^22^ was trained completely end-to-end for a fair comparison. We leveraged multi-phase MRI to improve vessel segmentations and investigated the optimal preprocessing pipeline for multi-phase organ segmentation on abdominal T1-VIBE MRI data. Furthermore, we evaluated the performance relative to distinct liver functions and provide a cross-scanner validation. Our automated approach replaces labor-intensive manual delineations or ROI placements with precise segmentations, supporting general surgical planning and providing detailed anatomical information that could aid future advancements in localized liver function assessment, ultimately improving post-operative survival estimation.

## Data

This retrospective, Health Insurance Portability and Accountability Act-compliant, single-center study was approved by the Ethics Committee at the University of Regensburg (IRB approval number: 23-3489-104). Written informed consent was waived due to the study’s retrospective nature, the use of de-identified data, and the minimal risk to patients, as no additional imaging or testing was performed. All procedures involving patient data adhered to the institutional and national research committees’ ethical standards and the principles outlined in the Declaration of Helsinki and its amendments.

This study comprises three distinct datasets, with their formation and interrelationships schematically illustrated in Supplementary Figure A1.

The patients from all datasets underwent Gd-EOB-DTPA-enhanced T1-weighted volumetric interpolated breath-hold examination (VIBE) MRI sequences with fat suppression during the native, arterial (AP), late arterial (LAP), portal venous (PVP), and hepatobiliary phases (HBP). The measured voxel size was 1.71 $\times$ 1.25 $\times$ 4.5 mm$^{3}$ and those were reconstructed to a voxel size of 1.25 $\times$ 1.25 $\times$ 3.0 mm$^{3}$. All images were obtained during a 14-second breath-hold, both before Gd-EOB-DTPA administration (native phase) and during the dynamic phases. Patients received an intravenous bolus injection of the contrast agent (0.025 mmol/kg body weight) at a flow rate of 1 mL/sec, followed by a 20 mL of 0.9% sodium chloride bolus for contrast-enhanced MRI.

**Dataset A** comprises a total of 458 unlabeled T1-weighted VIBE MRI acquisitions from patients with liver diseases. The inclusion criteria were: subjects aged over 18, no allergies to Gd-EOB-DTPA, availability of current liver function tests (blood or breath tests), images of passable quality, and no contraindications for MRI. This large set served as a comprehensive basis for optimizing our preprocessing pipeline, especially regarding the co-registration.

**Dataset B** is derived from **A** and serves as a labeled subset primarily used for training and evaluating segmentation performance. It consists of 78 labeled MRIs from patients with liver diseases. Initially, this dataset consisted of 59 patients with good image quality and typical lesion behavior. The additional inclusion criteria were a good image quality and current liver function evaluation via a $^{13}$C-Methacetin breath test (MBT), also called LiMAx test, prior or after a maximum of 5 days.

For core evaluation of the segmentation performance, a 4-fold split was applied to those 59 subjects. The schematic representation in supplementary Figure A1 illustrates how subsets within **B** were formed for training-validation splits during segmentation performance evaluation. In each dataset, one subset served as a test set while the remaining three subsets were used for training and validation. For example, in the first dataset, subsets 1–3 were used for training-validation (B1$_{\mathbf {train/val}}$) while subset 4 served as the test set (B1$_{\textbf{test}}$); this process was repeated until all subsets had been used as test sets. To enhance training robustness and model generalizability, 19 additional MRI acquisitions with substantial artifacts and atypical lesion contrast behavior were incorporated into all training-validation sets across the four folds. This helps to make it harder for the network to learn and to simulate real-world variability in clinical imaging. Therefore, three of the folds have 14 images in the test set and 64 images for training and validation, whereas the remaining fold has 17 subjects for evaluation and 61 for training and validation.

The images of both aforementioned datasets were acquired on a Siemens Magnetom Skyra 3 Tesla (T) MRI scanner, which is primarily used for those liver specific sequences in our clinic.

**Dataset C** served as a Cross-scanner validation dataset and contained 47 labeled T1-VIBE acquisitions from different MRI scanner types with 1.5T (Siemens Magnetom Sola, Siemens Magnetom Avanto Fit), explicitly excluding the 3T Siemens Magnetom Skyra used for Datasets **A** and **B**. The inclusion of different scanner types was intentional to introduce variability in scanner technology, simulating real-world conditions encountered in external institutions. This approach allowed for a broader evaluation of model generalizability across diverse hardware configurations. While these images were collected within the same clinical setting, differences in signal-to-noise ratios, contrast dynamics, and artifact profiles between scanners provided a realistic test for the robustness of the segmentation models. The inclusion criteria for **C** were: subjects aged over 18 years, no allergies to Gd-EOB-DTPA, and no contraindications to MRI. To reflect real-world imaging scenarios, this dataset contains both high-quality images and images of poor quality with artifacts. This diversity enables evaluation not only on optimal samples but also on challenging cases, further testing the robustness of the segmentation models.

## Methods

### MRI data preprocessing

The images of all five phases (native, arterial (AP), late arterial (LAP), portal venous (PVP), and hepatobiliary phases (HBP), acquired during Gd-EOB-DTPA-enhanced T1-VIBE sequences as per clinical protocols) for each patient were pre-processed with following pipeline in a Python v.3.10 environment:

1. Conversion from DICOM to NIFTI file format using dcm2niix v1.0.20211006^23^
2. Bias correction using the N4ITK algorithm^24^
3. Image-wise z-score normalization with numpy v.1.26.4^25^
4. Co-registration of all phases to the late arterial phase with the nipype interface (v.1.8.6) and ANTs registration (v.2.4.4) using three transformations in the order of rigid, affine and symmetric diffeomorphic registration (SyN)^26,27^

**Fig. 1:**
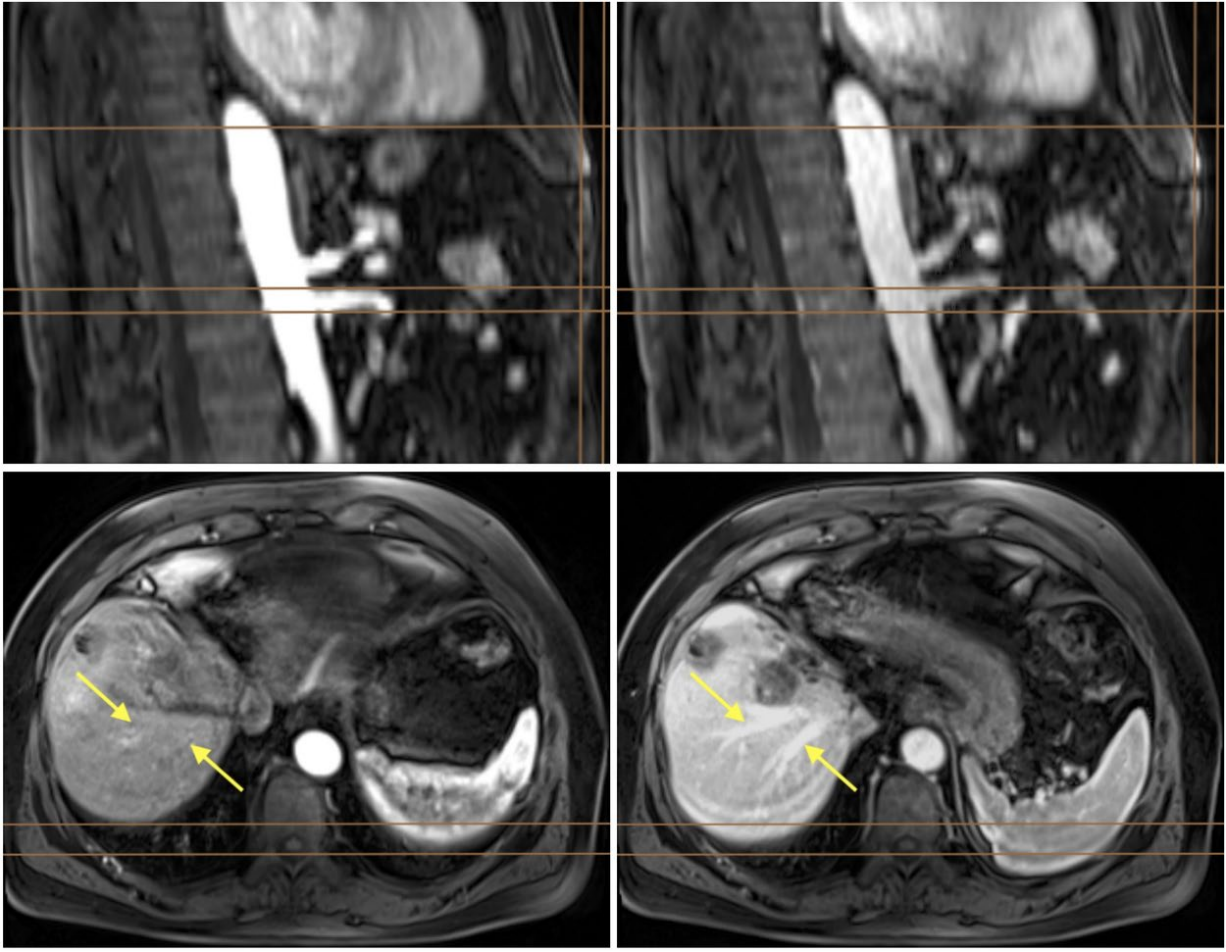
Arterial and portal venous phases from the T1-VIBE sequence for one subject before co-registration. The arterial phase is shown in the (left) images, and the portal venous phase in the (right). The (top row) displays the sagittal view. The (bottom row) shows the axial orientation of the same MRI. Images in the same row depict the exact same section. Due to variations in the breathing cycle, the abdominal organs do not remain in a fixed position. Brown reference lines aid visualization. Vertical lines in the top images indicate the moving abdominal border: the right line marks the border in the arterial phase, while the left line marks the border in the portal venous phase, where less air was exhaled. Horizontal lines in these images illustrate the movement of the heart (top line) and vessels (bottom lines) between phases due to breathing. Yellow arrows in the axial images point to identical spatial positions where the hepatic veins are visible in the portal venous phase. The horizontal lines indicate the borders of the liver and spleen during the arterial phase and demonstrate how they shift due to breathing.

In abdominal imaging with breath-hold techniques, specific challenges arise. Not all patients can exhale uniformly across all acquisitions. Unlike brain MRIs, where anatomical structures in different phases may vary in orientation while their shape is maintained, abdominal imaging experiences significant alterations due to diaphragm movement during exhalation, affecting organ positioning relative to each other and leading to deformation. An example of this is illustrated in Fig. 1, showing the same sagittal and axial sections from both arterial and portal venous phases of the T1-VIBE sequence. The images reveal notable differences in organ positioning between phases, complicating accurate co-registration of fine vascular structures like hepatic veins due to varying exhalation levels among patients. Those characteristics made the co-registration a challenging task. Therefore, we evaluated which phase is suited best as the fixed image for this step. Co-registration quality was measured by metrics such as the Mean Squared Error, Mutual Information, Structural Similarity Index, and the Normalized Cross Correlation, as well as by visual inspection.

The data from datasets **B** and **C** was labeled by one radiologist with more than 12 years of experience in liver diagnosis. This included the manual segmentation of the liver parenchyma, hepatic veins, portal veins, lesions, abdominal aorta, thoracic aorta and ascites in the preprocessed MRIs with the software ImFusion Labels v.0.21.5^28^. Subsequently, the labeled data was resliced into an isotropic voxel spacing of 1.2mm$^3$, resulting in an image size of [160x333x333] voxels.

While it may seem unconventional, our approach aimed to segment the plain liver parenchyma, deliberately excluding any kind of lesions or vascular structures. This design choice was intended to have a more comprehensive and automated method than the region-of-interest (ROI) placement methodology described in the studies referenced in the Introduction. Unlike traditional liver segmentation studies that focus primarily on hepatocellular carcinoma (HCC), we adopted a broader perspective, sub-classifying lesions into categories such as HCC, cholangiocarcinoma (CCC), focal nodular hyperplasia (FNH), adenoma, cysts, ablation defects, metastases, hemangiomas, biliomas, and regenerative nodules. However, because of the small dataset size, most of the lesions only occured one to three times and thus it was not always possible to include one case of each lesion type in the training-, validation- and test partitions. Combined with the small volumetric size of the majority of them compared to the other areas of interest, many were misclassified during test runs and thus they were combined into the class *lesions*.

### nnU-Net framework

The nnU-Net is a self-configuring deep learning framework for image segmentation that takes care of network configuration and also applies preprocessing steps to the MRI data, if required. As the dataset has already been preprocessed to have isotropic voxel spacings and images were z-score normalized, the framework didn’t apply any of those steps.

At the beginning of this study, the baseline architecture of the 3D fullres configuration in the nn-UNet framework was a plain 3D U-Net, as introduced in 2016 as an extension of the original U-Net from Ronneberger in 2015^5,6,29^.

In 2024, the baseline configuration changed to a 3D U-Net with residual blocks^30^ in the encoder stage and can occupy more VRAM. Therefore, the input patches can be larger^21^. In our study this led to a VRAM allocation of 8.5 GB for the standard nnU-Net and 28 GB for the ResEnc nnU-Net.

We completely trained and evaluated both configurations in a nested cross-validation (see Figure A1).

Unlike in the original U-Net, the ReLU activation function^31^ was replaced by leaky ReLU functions^32^ and instance normalization^33^ was used. Additionally, instead of max-pooling, strided convolutions were deployed.

For our datasets, the input patches for the 2024 baseline configuration were of size [112x256x256], whereas the initial baseline configuration was configured to have the dimensions of [80x192x160] for the extraced patches that are fed into the CNN. Both configurations were trained with a batch size of 2 and started with 32 filters in the first encoder stage. Those doubled for the next three stages, up to 256 filters. This was followed by two more encoding stages, both with a filter count of 320. As typical for all U-Net architectures, the amount of those was symmetrical in the corresponding encoder-decoder stages.

For the nnU-Net, an epoch is defined as a total of 250 batches and the amount of epochs is fixed to 1000.

The data from each of the four aforementioned folds in the data section, available to the training phase, was split into five different subsets that were used for training and validation by the nn-UNet^6,21^. The ability to train on five different folds on a training/validation dataset is implemented into the framework. In each fold, one of the subsets was used for validation, the other four were used for training. Performing this nested cross-validation (see Figure A1) resulted in 20 CNNs, each trained on a different training/validation dataset.

This was performed once for both baseline models, hence resulting in 40 end-to-end trained networks from the nnU-Net framework that will be publicly available after publication and can also be used for ensembled predictions.

The framework always chooses the 1000$^\text {th}$ epoch for inference by default, since the validation was only performed on a subset of 50 batches and therefore the validation loss generally is not representative to pick the model based on it.

### Swin UNETR

For this study, Swin UNETR was used as a well researched hybrid transformer model with a standardized and easy to use implementation within the MONAI framework^34^. The model was configured to use five input channels for the different modalities and an input image size of [128x128x128] voxels. The initial feature size of 48 doubled in each of the four stages in the encoding path. To ensure a fair comparison with the nnU-Net variants and the memory limitation of 40 GB VRAM, the model was trained with a batch size of 2 for a total of 1300 epochs. Furthermore, training and evaluation was performed with the exact same nested cross-validation.

In contrast to the nnU-Net framework, the validation was performed on all batches of the validation dataset and therefore the model with the lowest validation loss was used for inference on the test data. There, a sliding window method with batch size of 4 is employed for efficiency.

The data augmentation strategies for all architectures were kept as consistent as possible to ensure a fair comparison between the architectures. However, the specific implementation details may have varied slightly due to the differences in their respective frameworks.

The architecture was trained and evaluated in the exact same nested cross-validation as described for the nnU-Net variants, hence, 20 end-to-end trained networks of this architecture will be publicly available after publication.

### Evaluation metrics

To assess the performance of the co-registration and the segmentation methods, a variety of metrics was used. Those will be shortly introduced here.

**Co-registration:** *Mutual Information (MI)* measures the statistical dependency between two images, making it suitable for multi-modal image registration. The values are not capped and depend on the specific method that is used for the calculations, therefore those are not comparable to other published values. However, it is suitable for this paper, as all values were calculated with the same method on the same dataset^35^. *Normalized Cross-Correlation (NCC)* quantifies the linear correlation between image intensities, where a value of one indicates perfect correlation. The normalization makes it suitable for comparing multi-modal image co-registration, where absolute intensity values may vary significantly^36^. *Structural Similarity Index (SSIM)* assesses image similarity based on luminance, contrast, and structure, even when the absolute intensity values differ between modalities. The values range from 0 to 1, with 1 indicating perfect similarity^37^. *Mean Absolute Error (MAE)* measures the average absolute differences between corresponding pixel intensities. Even though it is very sensitive to large differences in intensity values and doesn’t account for any structural or perceptual differences in the images, it was still used as it is easy to understand and offers a more comprehensive evaluation of the co-registration performance in combination with the other metrics. Smaller values indicate better similarity^38^.

**Segmentation:** The *Dice Similarity Coefficient (DSC)* measures the overlap between predictions and ground truth segmentations, where true positives are double-weighed^39,40^. The closely related *Intersection over Union (IoU)* measures the overlap ratio between predicted and ground truth segmentations^41^. The DSC tends to produce higher values than IoU for the same segmentation result, due to its double-weighing of true positives in its calculation, making it more forgiving of errors in segmentation compared to the IoU. *Positive Predictive Value (PPV)* and *True Positive Rate (TPR)* assess precision and sensitivity, respectively. Lesion-wise- *True Poisitive Rate (LTPR)* and *False Positive Rate (LFPR)* evaluate the detection performance at the lesion level and is extremely valuable when dealing with small lesions, as those affect the metric as much as big lesions, which is not the case for the other metrics. *Volume Difference (VD)* quantifies the relative difference between the predicted and true segmented volumes.

## Results

### Co-registration

The co-registration of MRI volumes from different phases of the T1-VIBE sequence was conducted on dataset **A** to ensure precise alignment for further analysis. This process was assessed through visual inspection and various quantitative metrics: Mutual Information (MI), Normalized Cross Correlation (NCC), Mean Absolute Error (MAE), and Structural Similarity Index (SSIM), with detailed explanations provided in the previous subsection. The results are summarized in Table 1.

**Table 1:** This Table illustrates the co-registration performance on the 458 MRIs for different phases from the T1-VIBE sequence as the fixed image.

Fixed image	Mutual information ${\uparrow }$	Normalized cross correlation ${\uparrow }$	Mean absolute error ${\downarrow }$	Structural similarity index ${\uparrow }$
Native	0.90 [0.90, 0.91]	0.91 [0.91, 0.92]	1370 [1305, 1435]	0.83 [0.82, 0.83]
Arterial	0.92 [0.91, 0.93]	0.91 [0.91, 0.91]	1096 [1034, 1158]	0.87 [0.86, 0.87]
Late arterial	**1.03 [1.02, 1.05]**	**0.95 [0.95, 0.95]**	**804 [749, 860]**	**0.87 [0.87, 0.88]**
Portalvenous	*1.02 [1.01, 1.04]*	*0.95 [0.94, 0.95]*	*822 [767, 878]*	*0.87 [0.87, 0.87]*
HBP	0.92 [0.91, 0.93]	0.92 [0.92, 0.93]	1095 [1028, 1162]	**0.87 [0.87, 0.88]**

All MRI Volumes for each patient were co-registered to the given phase as the fixed image. Best and second best results are bold and italic, respectively. Numbers in brackets denote the 95%-confidence intervals for each metric.

Using the late arterial and portal venous phases as reference images showed superior performance across all metrics. The late arterial phase recorded the highest MI (1.03) and NCC (0.95), the lowest MAE (804), and a high SSIM (0.87). The portal venous phase also performed well, achieving an MI of 1.02, NCC of 0.95, MAE of 822, and SSIM of 0.87, indicating that the late arterial phase is optimal for co-registration. In contrast, co-registration with the native phase yielded lower results: MI of 0.90, NCC of 0.91, MAE of 1370, and SSIM of 0.83, suggesting it has less shared information and greater intensity differences due to varying contrast. Visual inspections corroborated these findings, confirming that the late arterial phase provided the best alignment. Consequently, the dataset where all phases were co-registered to the late arterial phase was selected for subsequent analyses.

### Segmentation

In this section, we present the segmentation performance of three deep learning architectures on data goups **A** and **B**, comprising seven labels. The models evaluated are the conventional nnU-Net, the ResEnc nnU-Net, and the Swin UNETR. The assessment was conducted with ensembled predictions to enhance robustness. The averaged results for the four test-sets (Figure A1) are displayed in Table 2.

**Table 2:** Segmentation performance of both nnU-Net configurations and the Swin UNETR for all seven labels with 5-fold cross validation and ensembled predictions.

nnU-Net^6^	DSC	IOU	PPV	TPR	LFPR	LTPR	VD
Liver parenchyma	**0.97 [0.97, 0.98]**	**0.95 [0.94, 0.96]**	**0.97 [0.96, 0.98]**	**0.98 [0.98, 0.99]**	–	–	**0.03 [0.02, 0.04]**
Portal vein	**0.83 [0.80, 0.87]**	**0.73 [0.69, 0.76]**	**0.86 [0.82, 0.89]**	**0.82 [0.78, 0.86]**	–	–	** 0.12 [0.08, 0.16]**
Hepatic veins	**0.78 [0.77, 0.80]**	**0.65 [0.63, 0.68]**	**0.83 [0.80, 0.85]**	0.77 [0.74, 0.80]	–	–	**0.18 [0.14, 0.22]**
Lesions	**0.56 [0.48, 0.64]**	**0.44 [0.36, 0.51]**	**0.78 [0.71, 0.86]**	**0.52 [0.44, 0.61]**	**0.10** **[0.04, 0.17]**	**0.65 [0.57, 0.74]**	**0.66 [0.38, 0.93]**
Ascites	**0.44 [0.16, 0.71]**	**0.32 [0.09, 0.55]**	*0.74 [0.42, 1.10]*	**0.36 [0.09, 0.63]**	–	–	*0.69 [0.25, 1.12]*
Abdominal aorta	**0.96 [0.96, 0.97]**	**0.93 [0.92, 0.94]**	**0.96 [0.96, 0.97]**	**0.97 [0.96, 0.98]**	–	–	**0.04 [0.03, 0.05]**
Thoracic aorta	**0.93 [0.91, 0.95]**	**0.87 [0.84, 0.90]**	**0.92 [0.89, 0.95]**	0.94 [0.93, 0.96]	–	–	**0.10 [0.07, 0.14]**
ResEnc nnU-Net^21^							
Liver parenchyma	**0.97 [0.97, 0.98]**	**0.95 [0.94, 0.96]**	*0.96 [0.95, 0.97]*	**0.98 [0.98, 0.99]**	–	–	*0.03 [0.02, 0.05]*
Portal vein	*0.83 [0.79, 0.86]*	*0.72 [0.68, 0.76]*	**0.86 [0.82, 0.89]**	*0.81 [0.77, 0.85]*	–	–	0.13 [0.09, 0.17]
Hepatic veins	*0.78 [0.76, 0.80]*	*0.64 [0.62, 0.67]*	**0.83 [0.80, 0.85]**	0.75 [0.72, 0.78]	–	–	*0.18 [0.15, 0.21]*
Lesions	*0.51 [0.43, 0.60]*	*0.40 [0.32, 0.48]*	*0.77 [0.69, 0.85]*	*0.47 [0.38, 0.56]*	*0.19* *[0.10, 0.27]*	0.63 [0.54, 0.72]	*0.73 [0.47, 0.98]*
Ascites	*0.41 [0.18, 0.64]*	*0.28 [0.11, 0.46]*	**0.77 [0.43, 1.11]**	*0.31 [0.09, 0.55]*	–	–	**0.67 [0.42, 0.91]**
Abdominal aorta	**0.96 [0.96, 0.97]**	**0.93 [0.92, 0.94]**	**0.96 [0.96, 0.97]**	0.96 [0.96, 0.97]	–	–	**0.04 [0.03, 0.05]**
Thoracic aorta	**0.93 [0.91, 0.95]**	**0.87 [0.84, 0.90]**	*0.92 [0.89, 0.94]*	**0.95 [0.93, 0.96]**	–	–	*0.12 [0.07, 0.16]*
Swin UNETR^22^							
Liver parenchyma	*0.96 [0.95, 0.97]*	*0.92 [0.90, 0.94]*	0.94 [0.92, 09.6]	*0.98 [0.97, 0.98]*	–	–	0.09 [0.05, 0.13]
Portal vein	0.74 [0.70, 0.78]	0.60 [0.56, 0.64]	*0.82 [0.78, 0.86]*	0.69 [0.64, 0.74]	–	–	0.35 [0.26, 0.43]
Hepatic veins	0.65 [0.61, 0.70]	0.51 [0.46, 0.55]	*0.78 [0.74, 0.81]*	0.61 [0.56, 0.66]	–	–	0.48 [0.38, 0.59]
Lesions	0.47 [0.40, 0.55]	0.35 [0.28, 0.41]	0.65 [0.56, 0.74]	0.44 [0.37, 0.52]	0.55 [0.46, 0.63]	*0.63* *[0.54, 0.73]*	1.47 [0.69, 2.25]
Ascites	0.28 [0.08, 0.49]	0.19 [0.04, 0.34]	0.45 [0.15, 0.74]	0.22 [0.05, 0.39]	–	–	3.99 [-2.76, 10.8]
Abdominal aorta	*0.94 [0.94, 0.95]*	*0.89 [0.88, 0.91]*	*0.95 [0.94, 0.96]*	0.94 [0.93, 0.96]	–	–	*0.11 [0.08, 0.14]*
Thoracic aorta	*0.87 [0.83, 0.90]*	*0.78 [0.74, 0.83]*	0.89 [0.86, 0.93]	0.86 [0.82, 0.91]	–	–	0.30 [0.21, 0.39]

Best and second best results are bold and italic, respectively. The 95%-confidence intervals are denoted in brackets.

**Fig. 2:**
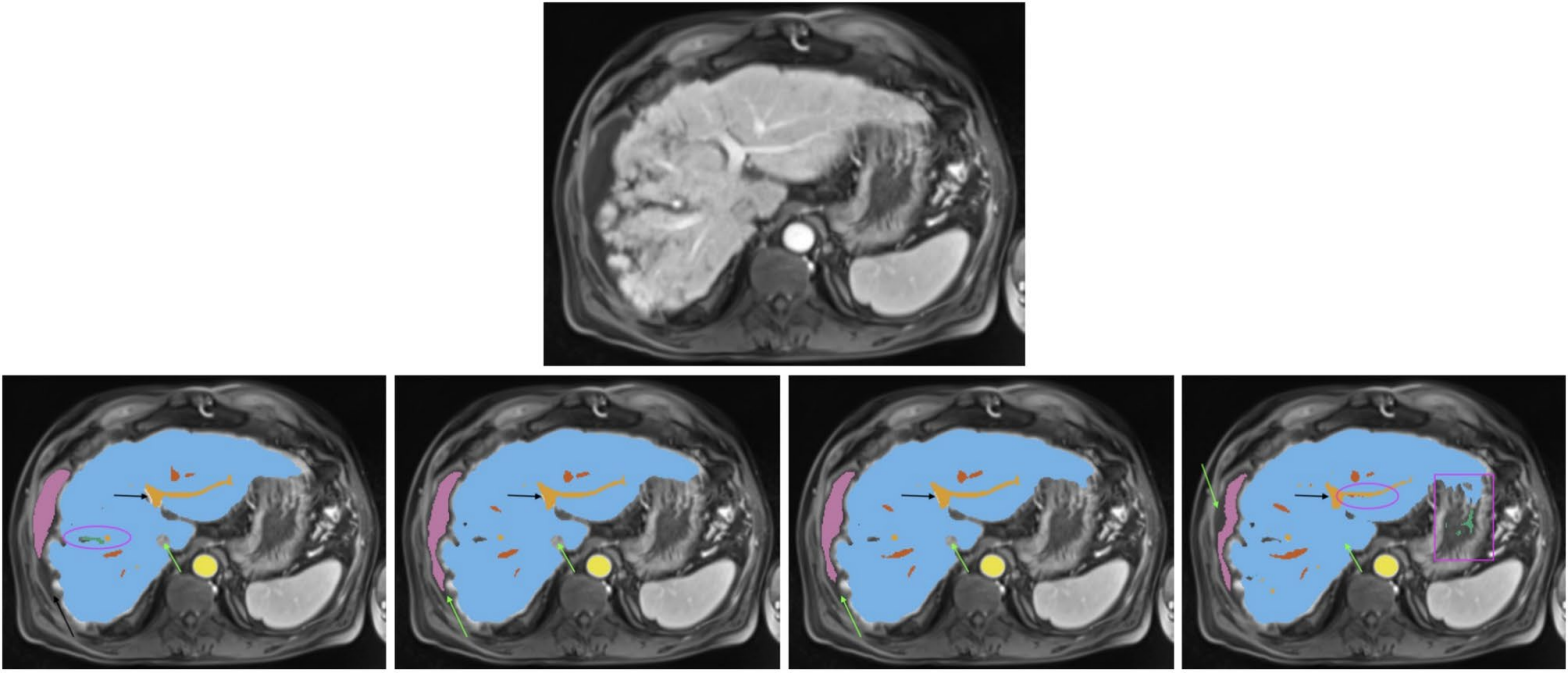
All images show the same axial section of a patient with liver cirrhosis and ascites for the late arterial phase. First row is the given image with no annotations. Labels in the other sections are blue (liver), orange (portal vein), red (hepatic vein), mauve (ascites), green (lesion), and yellow (abdominal aorta). The bottom row from left to right depicts the ground-truth annotations, standard nnU-Net’s, ResEnc nnU-Net’s and SWIN UNETR’s segmentations. Arrows, ellipses and rectangular boxes highlight major differences between the segmentations from the models and the ground-truth annotations.

*Liver Parenchyma Segmentation* Both nnU-Net variants demonstrated the best performance for liver parenchyma segmentation in this comparison, achieving high scores in DSC, IOU, and TPR. The conventional nnU-Net slightly outperformed its ResEnc counterpart in PPV and VD, indicating marginally superior precision and volume accuracy. While Swin UNETR produced competitive results, it lagged behind both nnU-Net variants across all metrics.

*Vessel Segmentation* For portal vein and hepatic vein segmentation, the conventional nnU-Net achieved the best performance across most metrics. The ResEnc nnU-Net showed comparable results but fell slightly behind in TPR for hepatic veins. Swin UNETR’s performance was notably lower, particularly in DSC, IOU, and TPR.

*Lesions and Ascites* Lesion segmentation proved challenging for all models, with the conventional nnU-Net achieving the highest DSC and IOU scores, as well as the lowest LFPR. The ResEnc variant followed closely, while Swin UNETR had the lowest scores across most metrics.

Similar observations were made for ascites segmentation. The conventional nnU-Net outperformed other models in DSC and IOU, closely followed by the ResEnc nnU-Net. Swin UNETR showed significantly lower performance. However, it’s worth noting that the values for this label should be interpreted cautiously. Visual inspection of the nnU-Net’s segmentations for this label revealed a notably high level of precision (see Fig. 2), which may not be fully captured by the numerical values alone. This discrepancy between quantitative measures and qualitative assessment underscores the complexity of evaluating segmentation performance.

*Aorta Segmentation* Both nnU-Net variants performed similarly well on abdominal and thoracic aorta segmentation, with the conventional nnU-Net slightly ahead in TPR. Swin UNETR, while competitive, was slightly behind in most metrics.

*Visual Analysis* A visual analysis of segmentation results for a patient with liver cirrhosis and ascites revealed that both nnU-Net variants correctly identified the vena cava inferior as non-liver tissue, whereas Swin UNETR misclassified it as liver (see Fig. 2). In ascites segmentation, the conventional nnU-Net provided the most comprehensive segmentation, followed by the ResEnc nnU-Net. Swin UNETR captured some areas missed by the other models but missed larger portions overall.

**Fig. 3:**
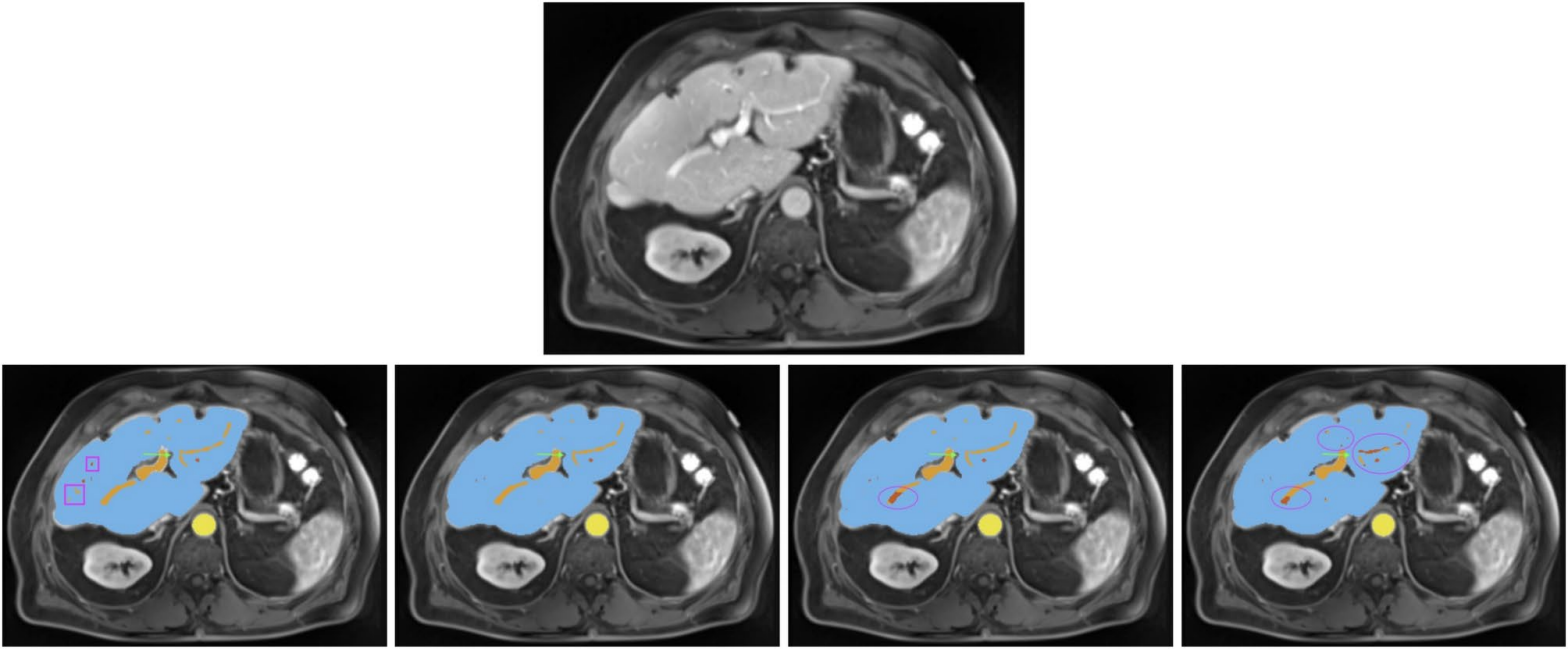
All images show the same axial section of a patient with liver disease for the portalvenous phase. First row is the given image with no annotations. Labels in the other images are blue (liver), orange (portal vein), red (hepatic vein) and yellow (abdominal aorta). The bottom row from left to right depicts the ground-truth annotations, standard nnU-Net’s, ResEnc nnU-Net’s and SWIN UNETR’s segmentations. Arrows and ellipses highlight major differences between the segmentations from the models and the ground-truth annotations. The pink rectangular boxes show hepatic and portal veins in the ground-truth annotations that all of the architectures missed.

For another patient with impaired liver function (see Fig. 3), the nnU-Net models demonstrated more precise liver parenchyma border segmentation compared to Swin UNETR. All models struggled with correct and precise segmentation of hepatic and portal veins in certain areas, where the ResEnc nnU-Net and the Swin UNETR even misclassified some portions of the portal vein as hepatic vein.

*Training duration* The training of the 20 networks per architecture took about 11, 32.5 and 10 days, for the standard nnU-Net, Residual Encoder nnU-Net and the Swin UNETR, respectively.

### Liver function-based segmentation analysis

In this subsection, the results presented in Table 2 are analyzed with respect to their different LiMAx scores. Specifically, the official LiMAx thresholds defined by Stockmann et al.^42^ were applied to categorize liver function into three groups: significant hepatic injury (LiMAx < 140; n = 13), limited hepatic impairment (140 $\le$ LiMAx < 314; n = 29), and normal liver function (LiMAx $\ge$ 315; n = 17). The comparison of segmentation performance, based on Dice scores, is summarized in Table 3. Additional details and a more comprehensive evaluation are provided in Supplementary Tables A1, A2, and A3.

*Normal liver function* All three architectures achieved high segmentation accuracy for liver parenchyma, abdominal aorta, and thoracic aorta, with DSC values consistently above 0.91. The standard nnU-Net slightly outperformed the other models in most cases, achieving a DSC of 0.98 for liver parenchyma and 0.96 for abdominal aorta. Portal vein segmentation also showed robust performance across models, with the standard nnU-Net achieving the highest DSC of 0.87. However, segmentation of lesions presented challenges, with lower DSC values ranging from 0.44 (ResEnc nnU-Net) to 0.56 (standard nnU-Net).

*Limited hepatic impairment* Segmentation accuracy remained high for larger anatomical structures such as liver parenchyma (DSC$\approx$0.97 across models) and abdominal aorta (DSC=0.96). However, performance decreased slightly for smaller structures like portal vein and hepatic veins, particularly for the Swin UNETR model (DSC=0.76 and 0.69, respectively). Lesion segmentation showed moderate accuracy across models, with the standard nnU-Net achieving the highest DSC of 0.56. Ascites detection was notably inconsistent, with DSC values ranging from 0.17 (Swin UNETR) to 0.35 (standard nnU-Net), reflecting the difficulty of segmenting this structure in T1-weighted images.

*Significant hepatic impairment* Segmentation performance declined further for smaller or more complex structures such as the portal vein and hepatic veins. The standard nnU-Net demonstrated relatively better robustness, achieving DSC values of 0.73 and 0.72, respectively, compared to Swin UNETR (0.63 and 0.46). Liver parenchyma segmentation remained strong across all models, with DSCs exceeding 0.95, highlighting the reliability of these architectures for larger structures even under severe impairment conditions. Lesion segmentation exhibited variability, with Swin UNETR achieving the lowest DSC (0.42) compared to 0.57 for the standard nnU-Net. Ascites detection showed improved performance in cases of significant hepatic injury, with DSC values exceeding 0.54. This improvement can be attributed to the fact that ascites is typically more pronounced in patients with worse liver function and tends to form a larger anatomical structure in such cases.

**Table 3:** Segmentation performance evaluated for different liver functions, based on the Dice Similarity Coefficient.

	standard nnU-Net^6^	ResEnc nnU-Net^21^	Swin UNETR^22^
Normal liver function	DSC		
Liver parenchyma	**0.98 [0.98, 0.98]**	0.98 [0.97, 0.98]	0.97 [0.97, 0.98]
Portal vein	**0.87 [0.84, 0.89]**	0.86 [0.84, 0.89]	0.77 [0.73, 0.81]
Hepatic veins	**0.81 [0.79, 0.84]**	**0.81 [0.79, 0.84]**	0.75 [0.70, 0.79]
Lesions	**0.56 [0.41, 0.72]**	0.44 [0.26, 0.63]	0.47 [0.31, 0.63]
Ascites	–	–	–
Abdominal aorta	**0.96 [0.95, 0.97]**	**0.96 [0.95, 0.97]**	0.94 [0.92, 0.96]
Thoracic aorta	0.91 [0.87, 0.95]	**0.91 [0.87, 0.96]**	0.85 [0.78, 0.92]
Limited hepatic impairment			
Liver parenchyma	**0.97 [0.96, 0.98]**	**0.97 [0.96, 0.98]**	0.95 [0.94, 0.97]
Portal vein	**0.86 [0.85, 0.87]**	0.85 [0.84, 0.87]	0.76 [0.72, 0.81]
Hepatic veins	**0.80 [0.78, 0.82]**	0.79 [0.76, 0.81]	0.69 [0.64, 0.73]
Lesions	**0.56 [0.44, 0.69]**	0.54 [0.41, 0.68]	0.49 [0.39, 0.60]
Ascites	**0.35 [0.00, 0.81]**	0.31 [0.00, 0.77]	0.17 [0.00, 0.44]
Abdominal aorta	**0.96 [0.96, 0.97]**	**0.96 [0.96, 0.97]**	0.95 [0.94, 0.96]
Thoracic aorta	**0.93 [0.89, 0.96]**	0.92 [0.89, 0.95]	0.87 [0.81, 0.94]
Significant hepatic impairment			
Liver parenchyma	**0.97 [0.96, 0.98]**	0.96 [0.95, 0.98]	0.95 [0.93, 0.97]
Portal vein	**0.73 [0.56, 0.89]**	0.71 [0.54, 0.88]	0.63 [0.46, 0.81]
Hepatic veins	0.72 [0.67, 0.76]	**0.72 [0.68, 0.77]**	0.46 [0.31, 0.60]
Lesions	**0.57 [0.37, 0.77]**	0.53 [0.33, 0.74]	0.42 [0.22, 0.62]
Ascites	**0.68 [0.42, 0.89]**	0.54 [0.35, 0.73]	0.57 [0.29, 0.84]
Abdominal aorta	**0.97 [0.96, 0.98]**	0.97 [0.96, 0.97]	0.95 [0.93, 0.96]
Thoracic aorta	**0.95 [0.94, 0.96]**	**0.95 [0.94, 0.96]**	0.88 [0.82, 0.94]

The results are demonstrated for the standard nnU-Net^6^, the ResEnc nnU-Net^21^ and the Swin UNETR^22^. Best results are written in bold.

The standard nnU-Net consistently outperformed ResEnc nnU-Net and Swin UNETR across most anatomical structures and liver function categories. While ResEnc nnU-Net achieved comparable results in many cases, it struggled slightly with lesion segmentation and smaller structures such as hepatic veins. Swin UNETR demonstrated lower accuracy overall, particularly for complex or small anatomical regions like the portal vein and lesions. Supplementary Tables A1, A2, and A3 provide additional insights into the strengths and weaknesses of each architecture across different liver function categories.

The findings emphasize that all three architectures perform well for major anatomical structures under normal liver function conditions. However, their performance diminishes in varying degrees under impaired liver function scenarios - especially for finer structures like the portal vein or hepatic veins, which become harder to delineate due to reduced visibility in imaging data. Among the evaluated models, the standard nnU-Net remains the most reliable choice overall, consistently achieving higher Dice scores across all categories and anatomical structures.

### Cross-scanner validation

The cross-scanner validation performed on dataset C (n=47) demonstrated the generalizability of our models across different MRI scanner architectures while revealing scanner-dependent performance variations. This dataset, acquired on 1.5T Siemens Magnetom Sola and Avanto Fit scanners, presented distinct challenges compared to the primary Skyra 3T data, including differences in signal-to-noise ratio, contrast dynamics, and artifact profiles. The conventional nnU-Net showed remarkable resilience to these variations, maintaining superior performance across most segmentation tasks in this comparison. The results are summarized in Table 4.

For liver parenchyma segmentation, all architectures maintained high Dice scores (nnU-Net: 0.97 [0.94, 0.99], ResEnc: 0.97 [0.95, 0.99], Swin UNETR: 0.92 [0.87, 0.97]), though a slight performance degradation became apparent in the transformer-based model.

The conventional nnU-Net demonstrated strong performance in vascular structure segmentation, achieving DSC values of 0.85 [0.81, 0.89] for portal vein and 0.83 [0.78, 0.88] for hepatic veins. The ResEnc nnU-Net exhibited reduced vascular precision (portal vein DSC: 0.76 [0.70, 0.81] vs. 0.83 [0.79, 0.86] internal), while Swin UNETR showed significant performance drops (hepatic vein DSC: 0.51 [0.44, 0.58] vs. 0.65 [0.61, 0.70] internal), indicating transformer architectures’ sensitivity to scanner-specific features.

Lesion segmentation performance varied notably across the three models. The conventional nnU-Net achieved the highest DSC (0.55 [0.43, 0.67]) and demonstrated strong precision (PPV: 0.93 [0.88, 0.98]). The lesion-wise true positive rate (LTPR) of 0.94 [0.87, 1.01] suggests that nearly all lesions were detected despite scanner differences, although the volume difference (VD) was higher than in the internal dataset. In contrast, the Swin UNETR showed lower performance (DSC: 0.25 [0.14, 0.36]) accompanied by a higher lesion-wise false positive rate (LFPR: 0.76 [0.67, 0.85]), indicating an increased rate of spurious detections.

Ascites segmentation proved challenging for all models when applied to the external validation dataset, with negligible DSC values. This may be attributed to the different contrast and intensity characteristics of ascitic fluid in 1.5T versus 3T scanners, as well as potential differences in sequence parameters that affect fluid visibility and shows where model generalization limits lie.

The aortic segmentation remained robust across scanner types, with the conventional nnU-Net achieving the best performance for both abdominal (DSC: 0.98 [0.98, 0.99]) and thoracic aorta (DSC: 0.97 [0.96, 0.99]), outperforming both ResEnc nnU-Net and Swin UNETR.

**Table 4:** Segmentation performance on the external validation set for all three architectures with ensembled predictions.

nnU-Net^6^	DSC	IOU	PPV	TPR	LFPR	LTPR	VD
Liver parenchyma	0.97 [0.94, 0.99]	0.94 [0.91, 0.97]	**0.98 [0.96, 1.00]**	0.96 [0.94, 0.99]	–	–	0.10 [0.02, 0.19]
Portal vein	**0.85 [0.81, 0.89]**	**0.76 [0.71, 0.81]**	**0.94 [0.92, 0.96]**	**0.80 [0.74, 0.85]**	–	–	**0.32 [0.23, 0.42]**
Hepatic veins	**0.83 [0.78, 0.88]**	**0.74 [0.68, 0.80]**	**0.93 [0.91, 0.95]**	**0.78 [0.72, 0.85]**	–	-	**0.34 [0.23, 0.45]**
Lesions	**0.55 [0.43, 0.67]**	**0.45 [0.32, 0.57]**	**0.93 [0.88, 0.98]**	**0.48 [0.34, 0.61]**	0.06 [0.00, 0.12]	**0.94 [0.87, 1.00]**	**0.90 [0.67, 1.12]**
Ascites	0.00 [0.00, 0.01]	0.00 [0.00, 0.01]	0.42 [0.00, 1.00]	0.00 [0.00, 0.00]	–	–	1.73 [0.00, 5.98]
Abdominal aorta	**0.98 [0.98, 0.99]**	**0.98 [0.98, 0.98]**	**0.98 [0.98, 0.99]**	**0.98 [0.97, 0.99]**	–	–	**0.01 [0.01, 0.02]**
Thoracic aorta	**0.97 [0.96, 0.99]**	**0.95 [0.94, 0.98]**	**0.99 [0.99, 0.99]**	**0.97 [0.96, 0.99]**	–	–	**0.03 [0.02, 0.04]**
ResEnc nnU-Net^21^							
Liver parenchyma	**0.97 [0.95, 0.99]**	**0.94 [0.92, 0.97]**	0.97 [0.95, 0.99]	**0.97 [0.96, 0.99]**	-	-	**0.09 [-0.00, 0.18]**
Portal vein	0.76 [0.70, 0.81]	0.64 [0.58, 0.70]	0.92 [0.88, 0.96]	0.69 [0.63, 0.75]	-	-	0.61 [0.33, 0.89]
Hepatic veins	0.76 [0.71, 0.82]	0.64 [0.58, 0.70]	0.90 [0.87, 0.93]	0.69 [0.63, 0.75]	-	-	0.46 [0.35, 0.57]
Lesions	0.37 [0.24, 0.50]	0.27 [0.15, 0.40]	0.87 [0.74, 1.00]	0.28 [0.15, 0.41]	0.12 [-0.01, 0.25]	0.82 [0.67, 0.96]	1.23 [1.02, 1.45]
Ascites	**0.02 [0.00, 0.21]**	**0.01 [0.00, 0.11]**	**0.47 [0.00, 1.00]**	**0.01 [0.00, 0.12]**	–	–	**1.70 [0.00, 5.80]**
Abdominal aorta	0.97 [0.96, 0.97]	0.94 [0.93, 0.95]	**0.98 [0.98, 0.99]**	0.95 [0.95, 0.96]	–	–	0.07 [0.06, 0.08]
Thoracic aorta	0.96 [0.94, 0.97]	0.92 [0.90, 0.94]	**0.99 [0.99, 1.00]**	0.93 [0.91, 0.95]	–	–	0.12 [0.08, 0.15]
Swin UNETR^22^							
Liver parenchyma	0.92 [0.87, 0.97]	0.88 [0.82, 0.93]	0.92 [0.88, 0.96]	0.93 [0.88, 0.98]	–	–	0.17 [0.04, 0.30]
Portal vein	0.61 [0.55, 0.67]	0.47 [0.41, 0.52]	0.71 [0.65, 0.77]	0.58 [0.52, 0.65]	–	–	0.70 [0.33, 1.07]
Hepatic veins	0.51 [0.44, 0.58]	0.37 [0.31, 0.43]	0.67 [0.60, 0.74]	0.47 [0.39, 0.54]	–	–	0.76 [0.59, 0.94]
Lesions	0.25 [0.14, 0.36]	0.18 [0.10, 0.27]	0.36 [0.23, 0.50]	0.28 [0.16, 0.40]	0.76 [0.67, 0.85]	0.77 [0.62, 0.92]	188 [0.00, 322]
Ascites	0.00 [0.00, 0.01]	0.00 [0.00, 0.00]	0.27 [0.00, 1.00]	0.00 [0.00, 0.00]	–	–	1.36 [0.00, 5.98]
Abdominal aorta	0.91 [0.89, 0.92]	0.83 [0.81, 0.86]	0.91 [0.89, 0.92]	0.92 [0.89, 0.94]	–	–	0.17 [0.11, 0.22]
Thoracic aorta	0.84 [0.79, 0.90]	0.77 [0.70, 0.83]	0.88 [0.85, 0.92]	0.85 [0.78, 0.92]	–	–	0.32 [0.19, 0.45]

Best results are bold and the 95%-confidence intervals are denoted in brackets.

These findings highlight the importance of model selection when deploying across heterogeneous scanner environments in clinical settings. The conventional nnU-Net architecture demonstrated superior generalizability in this comparison, maintaining consistent performance levels comparable to those observed in the internal validation, for most structures.

## Discussion

We present fully end-to-end trained models for the automated segmentation of the liver and adjacent structures and make them available to the public. By employing a multi-phase approach, our model shows competitive performance even for complex structures such as hepatic vasculature, while contending with the additional challenges posed by co-registration. The whole pipeline can serve as a foundation for further research on liver diseases and their therapy.

We chose the nnU-Net architecture for a multitude of reasons. Its advantages include the fully automated self-configuring workflow, its adaptiveness that optimizes the architecture to any given image input, and its proven robustness and efficacy in medical segmentation tasks. For example, it is the core of the TotalSegmentator, a widely used tool for multi-label whole-body CT segmentation and just recently added functionality for MRI as well^43,44^. Furthermore, the framework is well-maintained and allows for easy sharing and integration of our trained models. The developed models, particularly the nnU-Net variants, demonstrate significant potential for integration into clinical workflows by automating segmentation tasks to aid preoperative planning, MRI-based liver function assessment, and treatment monitoring. With this, diagnostic precision can be enhanced and the workload for radiologists reduced. The high generalizability of the nnU-Net across scanner types further underscores its suitability for multi-center deployment, paving the way for standardized and efficient liver imaging analysis.

Furthermore, the claims of superior performance of novel architectures don’t seem to hold up, as a recent comprehensive benchmark shows. Isensee et al. reported that the nnU-Net performs competitively, even compared to the latest models like the SwinUNETRV2 and U-Mamba, which are based on the transformer and mamba architectures, respectively^7,8,21^. Our findings support those statements and highlight the importance of model selection when deploying across heterogeneous scanner environments in clinical settings. The conventional nnU-Net architecture demonstrated superior generalizability, maintaining consistent performance levels comparable to those observed in the internal validation, for most structures. This robustness makes it particularly suitable for multi-center applications or implementations across varied hardware configurations. In contrast, the Swin UNETR, despite its theoretical advantages in modeling long-range dependencies, appeared more susceptible to variations in image acquisition parameters and scanner characteristics, suggesting that transformer-based architectures may require more scanner-specific fine-tuning or domain adaptation strategies to achieve optimal performance in diverse clinical environments.

In an objective comparison using some of the most popular medical segmentation datasets, CNN-based U-Nets performed best. MedNeXt ranked first, closely followed by the baseline nnU-Net^21,45^. However, considering the significantly lower computing time required by the nnU-Net, the slight difference in reported performance is negligible. The authors argue that the trend towards novel architectures is in part based on the lack of rigorous validation, leading to bias and unsubstantiated claims of superiority. Our study addresses this issue and even goes one step further. Unlike studies that rely on a fixed test set, which can introduce bias due to lack of representativeness, our nested cross-validation ensures that every data point is used for testing exactly once. In addition to nested cross-validation, we performed cross-scanner validation to further ensure generalizability across diverse imaging conditions.

A stratified analysis based on LiMAx scores revealed that while liver parenchyma segmentation is robust across all liver function levels, accuracy for vascular segmentation declines with worsening hepatic impairment. Additionally, cross-scanner validation on 1.5T MRI data confirmed the clinical transferability of our approach, despite some scanner-dependent differences. This dual validation framework underscores the robustness and unbiased nature of our evaluation of the model performance.

Building on this technical foundation, it is important to contextualize these findings within the broader landscape of liver segmentation research. Nowadays, liver segmentation is mainly limited to segmenting the whole organ and its tumors. Moreover, models based on CT images are overrepresented. This trend can be attributed to the broad availability of public image datasets as part of segmentation challenges^46^. Among the most popular challenges is the Liver Tumor Segmentation Benchmark (LiTS), which encompasses 201 abdominal CT scans, the majority of which contain liver lesions^47^. It is part of the Medical Segmentation Decathlon that tests how well methods can generalize to previously unseen tasks and is widely used for benchmarking^48^. While the Medical Segmentation Decathlon includes a hepatic vessel segmentation task, it relies solely on CT images. This leaves MRI, particularly multi-phase MRI, significantly underrepresented in liver segmentation datasets. This gap is noteworthy, given that MRI offers a safer alternative to CT by avoiding harmful radiation exposure. MRI plays a crucial role in detecting, differentially diagnosing, and monitoring various liver diseases, and has shown promising results in assessing liver function^9–12,14,49^. However, those approaches require manual placement of regions of interest (ROIs) into several desired tissues, a limitation that our pipeline with its trained models aims to overcome.

Other approaches exclusively perform hepatic vessel segmentation^19,20,50^. Hence, work more similar to ours that reports multi-label performance on larger (e.g., liver parenchyma) as well as more complex structures (e.g., hepatic vessels) is rare. A paper that is somehow comparable to our approach employs a 3D residual U-Net that was trained on contrast-enhanced T1-weighted MRI in the hepatobiliary phase from 120 patients^18^. The authors report DSCs for the liver parenchyma (0.92 ± standard deviation of 0.03), tumor mass (0.77 ± 0.21), hepatic vein (0.70 ± 0.05), portal vein (0.61 ± 0.03) and bile duct (0.58 ± 0.15). However, this study only used a single phase from the T1-VIBE sequence. In contrast, our approach, despite some co-registration challenges, achieved higher DSCs for the hepatic and portal veins. This underscores the importance of incorporating multiple contrast phases to enhance segmentation performance. We leverage the individual strengths of each contrast phase in the visualization of different hepatic structures. The better performance in tumor segmentation, as reported by Oh et al., may be due to the differences in the patient collectives. The authors only included preoperative MRI of patients with hepatocellular carcinoma with a median size of 2.5 cm in the validation set. In contrast, we included a much more diverse selection of hepatic lesions (benign and malignant), many of which were post-interventional ablation defects. We further include segmentations of the thoracic and abdominal aorta that can help in applications where the relative enhancement compared to the contrast agent bolus plays a role.

## Limitations

First, the need for co-registration in multi-phasic MRI was not always optimal. The approach, which is adapted from neuroimaging, is complicated in our use case by the greater (relative) mobility (e.g., due to breathing) and elastic deformability of depicted structures. However, visual verification of the results and good performance metrics support our approach. Future studies could benefit from co-registration algorithms developed specifically for abdominal imaging that better account for respiratory motion and organ deformities.

Second, the relatively small dataset posed challenges for training deep learning models. Although the sophisticated data augmentation process within the nnU-Net framework – adapted to the Swin UNETR in this study – helped mitigate this issue, dataset limitations still impacted performance. Specifically, the small number of cases with ascites and different lesion types likely contributed to suboptimal segmentation results for these regions.

Visual inspection in dataset **B** revealed instances where the model’s segmentations for ascites deviated from the established ground truth but were considered clinically plausible by expert radiologists. These observations, despite lower calculated metrics, reflect the inherent challenges of using single-rater annotations as ground truth in tasks where inter-rater variability is significant. Such variability underscores that there is often no single “perfect” delineation, particularly in complex cases. These findings highlight the need for further studies incorporating multi-rater consensus annotations to better capture variability and ensure robust evaluation of model performance. Including T2-weighted sequences, where ascites appears hyperintense, could have been highly beneficial to improve agreement in these areas of interest, as well. Unfortunately, this was not feasible due to significant differences in spacing and field of view between T2- and T1-weighted acquisitions in our datasets.

Future studies should aim to standardize acquisition protocols to enable the integration of additional imaging modalities that could enhance segmentation accuracy.

Similarly, the segmentation of lesions was also affected by the dataset constraints. Performance could potentially be improved by either increasing the overall dataset size or narrowing the diversity of lesions to be segmented for specific applications. This would allow models to focus on fewer lesion types, improving their accuracy and generalization within these subgroups.

## Conclusion

Our study demonstrates that the traditional convolutional architecture of nnU-Net remains highly effective for medical image segmentation. Transformer-based models like Swin UNETR, while promising, often fall short of the performance achieved by CNN-based models, especially with datasets comprising multiple labels and in our case, if there are cross-scanner variabilities introduced.

Building on existing research, we reaffirm nnU-Net as one of the top-performing models across diverse segmentation tasks. We have developed and made publicly available (after publication) a complete pipeline with fully trained models for multi-label segmentation of the liver and adjacent structures in T1-VIBE MRIs. This provided data offers flexibility, allowing users to either implement the entire pipeline or integrate trained models into existing custom workflows.

This work not only advances the field of liver segmentation but also lays a robust foundation for developing more sophisticated models capable of anatomical segmentation, predicting liver lesion histopathology, and assessing liver function, with transformative potential for clinical practice and hepatology research.

## Supplementary Information

Supplementary Information.

## Data Availability

The datasets generated and analyzed during the current study are not publicly available due to the fact that they contain protected health information but are available from the corresponding author on reasonable request. The full code implementation, including instructions for deployment and all trained models, will be publicly available at https://github.com/FlorianRaab95 after publication.
