# Supplementary material for: Automatic segmentation of liver structures in multi-phase MRI using variants of nnU-Net and Swin UNETR
Source: Sci Rep. 2025 Jul 16;15:25740. doi: 10.1038/s41598-025-07084-5 (PMC12267559; doi:10.1038/s41598-025-07084-5)
Supplement: Supplementary file 1 — Supplementary Information. [file 41598_2025_7084_MOESM1_ESM.pdf]

Automatic Segmentation of Liver Structures in Multi-Phase MRI Using Variants of nnU-Net and Swin UNETR

Florian Raab[1,2,3*], Quirin Strotzer[3*], Christian Stroszczynski[3], Claudia Fellner[3], Ingo Einspieler[3], Michael Haimerl[3,4] Elmar W. Lang[2]
[1]Physics Department, University of Regensburg, Regensburg, 93053, Germany
[2]Biophysics Department, CIML Group, University of Regensburg, Regensburg, 93053, Germany
[3]Radiology Department, University Hospital Regensburg, Regensburg, 93053, Germany
[4]Radiology Department, Klinikum Würzburg Mitte, Würzburg, 97074, Germany

Supplementary Information

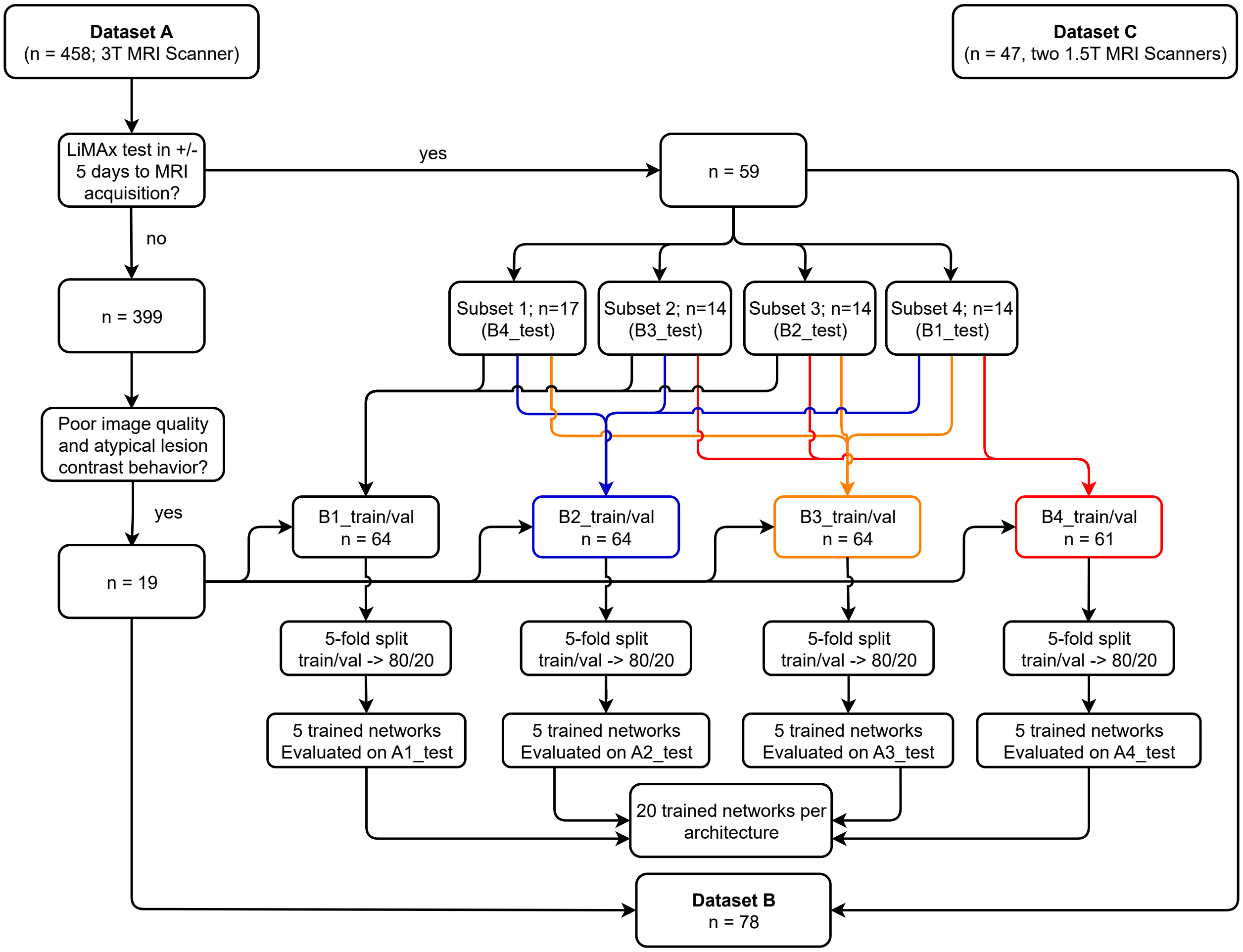

Figure A1. Schematic representation of how the Datasets for this study are formed.

Table A1. Segmentation performance of all three architectures with ensembled predictions on subjects with significant hepatic injury (LiMAx < 140). 95%-confidence intervals are denoted in brackets.

nnU-Net[1]	DSC	IOU	PPV	TPR	LFPR	LTPR	VD
**Liver parenchyma**	0.97 [0.96, 0.98]	0.94 [0.93, 0.96]	0.96 [0.94, 0.98]	0.98 [0.97, 0.99]	-	-	0.05 [0.02, 0.09]
**Portal vein**	0.73 [0.56, 0.89]	0.62 [0.45, 0.78]	0.77 [0.61, 0.94]	0.70 [0.53, 0.88]	-	-	0.46 [0.15, 0.77]
**Hepatic veins**	0.72 [0.67, 0.76]	0.56 [0.50, 0.62]	0.81 [0.73, 0.89]	0.68 [0.59, 0.76]	-	-	0.49 [0.28, 0.70]
**Lesions**	0.57 [0.37, 0.77]	0.44 [0.27, 0.61]	0.66 [0.42, 0.90]	0.55 [0.35, 0.75]	0.25 [0.02, 0.52]	0.53 [0.31, 0.75]	1.68 [0.00, 3.83]
**Ascites**	0.55 [0.00, 1.36]	0.42 [0.00, 1.13]	0.95 [0.90, 1.00]	0.44 [0.00, 1.00]	-	-	0.93 [0.00, 2.26]
**Abdominal aorta**	0.97 [0.96, 0.98]	0.94 [0.93, 0.95]	0.97 [0.96, 0.98]	0.97 [0.96, 0.98]	-	-	0.04 [0.03, 0.05]
**Thoracic aorta**	0.95 [0.94, 0.96]	0.91 [0.89, 0.92]	0.96 [0.93, 0.98]	0.95 [0.92, 0.97]	-	-	0.11 [0.07, 0.15]
**ResEnc nnU-Net[2]**							
**Liver parenchyma**	0.97 [0.95, 0.98]	0.94 [0.91, 0.96]	0.95 [0.92, 0.98]	0.98 [0.97, 0.99]	-	-	0.08 [0.02, 0.14]
**Portal vein**	0.71 [0.54, 0.88]	0.60 [0.43, 0.76]	0.77 [0.59, 0.94]	0.68 [0.51, 0.86]	-	-	0.49 [0.17, 0.82]
**Hepatic veins**	0.72 [0.68, 0.77]	0.57 [0.51, 0.63]	0.80 [0.73, 0.87]	0.68 [0.60, 0.76]	-	-	0.42 [0.30, 0.54]
**Lesions**	0.54 [0.33, 0.74]	0.41 [0.24, 0.58]	0.67 [0.42, 0.91]	0.50 [0.28, 0.72]	0.26 [0.01, 0.53]	0.50 [0.30, 0.70]	1.74 [0.00, 3.74]
**Ascites**	0.54 [0.35, 0.73]	0.37 [0.20, 0.55]	0.98 [0.90, 1.00]	0.38 [0.20, 0.56]	-	-	1.06 [0.72, 1.40]
**Abdominal aorta**	0.97 [0.96, 0.97]	0.94 [0.93, 0.95]	0.97 [0.96, 0.98]	0.97 [0.95, 0.98]	-	-	0.05 [0.04, 0.07]
**Thoracic aorta**	0.95 [0.94, 0.96]	0.91 [0.89, 0.92]	0.96 [0.93, 0.98]	0.95 [0.92, 0.97]	-	-	0.10 [0.05, 0.15]
**Swin UNETR[3]**							
**Liver parenchyma**	0.95 [0.93, 0.97]	0.91 [0.87, 0.94]	0.93 [0.90, 0.97]	0.97 [0.96, 0.98]	-	-	0.08 [0.03, 0.13]
**Portal vein**	0.63 [0.46, 0.81]	0.51 [0.35, 0.67]	0.79 [0.61, 0.97]	0.56 [0.39, 0.73]	-	-	0.57 [0.23, 0.91]
**Hepatic veins**	0.46 [0.31, 0.60]	0.32 [0.20, 0.45]	0.78 [0.68, 0.89]	0.39 [0.23, 0.54]	-	-	0.97 [0.65, 1.28]
**Lesions**	0.42 [0.22, 0.62]	0.31 [0.15, 0.47]	0.54 [0.27, 0.80]	0.43 [0.24, 0.63]	0.59 [0.34, 0.83]	0.46 [0.26, 0.66]	3.35 [0.00, 7.28]
**Ascites**	0.57 [0.29, 0.84]	0.40 [0.14, 0.66]	0.87 [0.64, 1.00]	0.43 [0.15, 0.71]	-	-	0.87 [0.29, 1.45]
**Abdominal aorta**	0.95 [0.93, 0.96]	0.90 [0.87, 0.93]	0.95 [0.93, 0.96]	0.94 [0.90, 0.98]	-	-	0.12 [0.05, 0.18]
**Thoracic aorta**	0.88 [0.82, 0.94]	0.79 [0.71, 0.87]	0.93 [0.89, 0.96]	0.85 [0.75, 0.94]	-	-	0.27 [0.14, 0.39]

Table A2. Segmentation performance of all three architectures with ensembled predictions on subjects with limited hepatic impairment ($140 \leq$ LiMAx < 315) with 5-fold cross validation. 95%-confidence intervals denoted in brackets.

nnU-Net[1]	DSC	IOU	PPV	TPR	LFPR	LTPR	VD
**Liver parenchyma**	0.97 [0.96, 0.98]	0.95 [0.93, 0.97]	0.96 [0.94, 0.98]	0.98 [0.98, 0.99]	-	-	0.06 [0.01, 0.10]
**Portal vein**	0.86 [0.85, 0.87]	0.75 [0.73, 0.78]	0.89 [0.87, 0.91]	0.84 [0.81, 0.86]	-	-	0.15 [0.11, 0.20]
**Hepatic veins**	0.80 [0.78, 0.82]	0.67 [0.64, 0.70]	0.85 [0.82, 0.88]	0.77 [0.73, 0.81]	-	-	0.28 [0.20, 0.35]
**Lesions**	0.56 [0.44, 0.68]	0.45 [0.34, 0.56]	0.78 [0.66, 0.90]	0.55 [0.42, 0.68]	0.09 [0.01, 0.16]	0.67 [0.54, 0.80]	1.10 [0.53, 1.67]
**Ascites**	0.35 [0.00, 0.81]	0.24 [0.00, 0.59]	0.59 [0.00, 1.00]	0.30 [0.00, 0.80]	-	-	1.37 [0.00, 3.00]
**Abdominal aorta**	0.96 [0.96, 0.97]	0.93 [0.92, 0.94]	0.96 [0.96, 0.97]	0.97 [0.95, 0.98]	-	-	0.06 [0.04, 0.09]
**Thoracic aorta**	0.93 [0.89, 0.96]	0.87 [0.83, 0.92]	0.92 [0.88, 0.97]	0.93 [0.90, 0.97]	-	-	0.16 [0.09, 0.23]
**ResEnc nnU-Net[2]**							
**Liver parenchyma**	0.97 [0.96, 0.98]	0.94 [0.92, 0.96]	0.96 [0.94, 0.98]	0.98 [0.98, 0.99]	-	-	0.07 [0.02, 0.11]
**Portal vein**	0.85 [0.84, 0.87]	0.75 [0.72, 0.77]	0.89 [0.87, 0.91]	0.83 [0.80, 0.85]	-	-	0.17 [0.12, 0.21]
**Hepatic veins**	0.79 [0.76, 0.81]	0.65 [0.62, 0.69]	0.85 [0.81, 0.88]	0.75 [0.71, 0.78]	-	-	0.30 [0.23, 0.36]
**Lesions**	0.54 [0.41, 0.68]	0.43 [0.32, 0.55]	0.76 [0.63, 0.89]	0.53 [0.40, 0.65]	0.17 [0.04, 0.30]	0.61 [0.47, 0.75]	1.21 [0.64, 1.79]
**Ascites**	0.31 [0.15, 0.77]	0.21 [0.15, 0.58]	0.62 [0.07, 1.00]	0.27 [0.27, 0.81]	-	-	1.22 [0.24, 2.21]
**Abdominal aorta**	0.96 [0.96, 0.97]	0.93 [0.92, 0.94]	0.96 [0.95, 0.97]	0.96 [0.95, 0.98]	-	-	0.07 [0.04, 0.09]
**Thoracic aorta**	0.92 [0.89, 0.95]	0.86 [0.82, 0.91]	0.92 [0.87, 0.96]	0.94 [0.91, 0.96]	-	-	0.19 [0.10, 0.29]
**Swin UNETR[3]**							
**Liver parenchyma**	0.95 [0.94, 0.97]	0.92 [0.89, 0.94]	0.93 [0.90, 0.96]	0.98 [0.98, 0.99]	-	-	0.12 [0.04, 0.19]
**Portal vein**	0.76 [0.72, 0.81]	0.63 [0.58, 0.68]	0.82 [0.77, 0.86]	0.73 [0.67, 0.78]	-	-	0.25 [0.16, 0.33]
**Hepatic veins**	0.69 [0.64, 0.73]	0.54 [0.49, 0.59]	0.77 [0.73, 0.82]	0.64 [0.59, 0.70]	-	-	0.36 [0.24, 0.49]
**Lesions**	0.49 [0.39, 0.60]	0.36 [0.27, 0.45]	0.67 [0.55, 0.79]	0.47 [0.36, 0.58]	0.55 [0.43, 0.67]	0.64 [0.50, 0.78]	1.11 [0.66, 1.56]
**Ascites**	0.17 [0.09, 0.44]	0.11 [0.07, 0.28]	0.28 [0.07, 0.64]	0.14 [0.11, 0.39]	-	-	6.33 [0.00, 20.96]
**Abdominal aorta**	0.95 [0.94, 0.96]	0.90 [0.87, 0.93]	0.95 [0.93, 0.96]	0.95 [0.93, 0.97]	-	-	0.11 [0.07, 0.15]
**Thoracic aorta**	0.87 [0.81, 0.94]	0.80 [0.73, 0.87]	0.89 [0.84, 0.95]	0.87 [0.79, 0.95]	-	-	0.27 [0.16, 0.39]

Table A3. Segmentation performance of all three architectures with and ensembled predictions on subjects with normal liver function(315 < LiMAx) with 5-fold cross validation. 95%-confidence intervals denoted in brackets.

nnU-Net[1]	DSC	IOU	PPV	TPR	LFPR	LTPR	VD
**Liver parenchyma**	0.98 [0.98, 0.98]	0.96 [0.96, 0.97]	0.98 [0.97, 0.99]	0.98 [0.98, 0.99]	-	-	0.03 [0.02, 0.04]
**Portal vein**	0.87 [0.84, 0.89]	0.77 [0.73, 0.80]	0.85 [0.82, 0.89]	0.88 [0.86, 0.91]	-	-	0.14 [0.07, 0.20]
**Hepatic veins**	0.81 [0.79, 0.84]	0.69 [0.65, 0.73]	0.81 [0.76, 0.86]	0.83 [0.79, 0.87]	-	-	0.25 [0.11, 0.38]
**Lesions**	0.56 [0.41, 0.72]	0.43 [0.28, 0.59]	0.86 [0.80, 0.92]	0.48 [0.31, 0.65]	0.04 [0.00, 0.10]	0.72 [0.56, 0.87]	0.79 [0.48, 1.11]
**Ascites**	-	-	-	-	-	-	-
**Abdominal aorta**	0.96 [0.95, 0.97]	0.92 [0.90, 0.94]	0.96 [0.94, 0.98]	0.96 [0.95, 0.98]	-	-	0.08 [0.04, 0.11]
**Thoracic aorta**	0.91 [0.87, 0.95]	0.85 [0.79, 0.91]	0.89 [0.83, 0.96]	0.95 [0.92, 0.98]	-	-	0.27 [0.09, 0.46]
**ResEnc nnU-Net[2]**							
**Liver parenchyma**	0.98 [0.98, 0.98]	0.96 [0.95, 0.97]	0.98 [0.97, 0.99]	0.98 [0.98, 0.99]	-	-	0.03 [0.02, 0.04]
**Portal vein**	0.86 [0.84, 0.89]	0.76 [0.72, 0.80]	0.86 [0.83, 0.90]	0.86 [0.83, 0.89]	-	-	0.14 [0.09, 0.19]
**Hepatic veins**	0.81 [0.79, 0.84]	0.69 [0.65, 0.73]	0.82 [0.77, 0.86]	0.83 [0.78, 0.87]	-	-	0.26 [0.13, 0.38]
**Lesions**	0.44 [0.26, 0.63]	0.35 [0.18, 0.52]	0.86 [0.78, 0.94]	0.37 [0.19, 0.56]	0.15 [0.04, 0.26]	0.75 [0.59, 0.91]	1.02 [0.70, 1.35]
**Ascites**	-	-	-	-	-	-	-
**Abdominal aorta**	0.96 [0.95, 0.97]	0.92 [0.91, 0.94]	0.96 [0.94, 0.98]	0.96 [0.95, 0.98]	-	-	0.07 [0.04, 0.11]
**Thoracic aorta**	0.91 [0.87, 0.96]	0.85 [0.78, 0.91]	0.88 [0.81, 0.95]	0.96 [0.92, 1.00]	-	-	0.30 [0.08, 0.52]
**Swin UNETR[3]**							
**Liver parenchyma**	0.97 [0.97, 0.98]	0.94 [0.93, 0.96]	0.97 [0.95, 0.98]	0.98 [0.97, 0.98]	-	-	0.04 [0.02, 0.06]
**Portal vein**	0.77 [0.73, 0.81]	0.63 [0.58, 0.69]	0.83 [0.78, 0.89]	0.73 [0.67, 0.80]	-	-	0.34 [0.24, 0.43]
**Hepatic veins**	0.75 [0.70, 0.79]	0.60 [0.54, 0.66]	0.80 [0.73, 0.86]	0.72 [0.66, 0.78]	-	-	0.36 [0.24, 0.47]
**Lesions**	0.47 [0.31, 0.63]	0.35 [0.21, 0.49]	0.67 [0.50, 0.83]	0.40 [0.25, 0.56]	0.50 [0.34, 0.66]	0.76 [0.60, 0.92]	0.77 [0.50, 1.04]
**Ascites**	-	-	-	-	-	-	-
**Abdominal aorta**	0.94 [0.92, 0.96]	0.89 [0.85, 0.92]	0.95 [0.93, 0.96]	0.94 [0.90, 0.98]	-	-	0.12 [0.05, 0.18]
**Thoracic aorta**	0.85 [0.78, 0.92]	0.76 [0.66, 0.85]	0.87 [0.77, 0.97]	0.86 [0.80, 0.92]	-	-	0.39 [0.14, 0.64]

References

1. Isensee, F., Jaeger, P. F., Kohl, S. A. A., Petersen, J. & Maier-Hein, K. H. nnu-net: a self-configuring method for deep learning-based biomedical image segmentation. *Nat. Methods* **18**, 203–211, DOI: 10.1038/s41592-020-01008-z (2021).

2. Isensee, F. *et al.* nnu-net revisited: A call for rigorous validation in 3d medical image segmentation (2024). 2404.09556.

3. Liu, Z. *et al.* Swin transformer: Hierarchical vision transformer using shifted windows. In *Proceedings of the IEEE/CVF International Conference on Computer Vision (ICCV)*, 10012–10022 (2021).

List of Figures in Appendix

A1 Flowchart of the Datasets 1

List of Tables in Appendix

A1 Segmentation performance of the three architectures on subjects with significant hepatic injury 2
A2 Segmentation performance of the three architecture on subjects with limited hepatic impairment 2
A3 Segmentation performance of the three architecture on subjects with normal liver function 3
